# Lipoprotein(a): Its Association with Calcific Aortic Valve Stenosis, the Emerging RNA-Related Treatments and the Hope for a New Era in “Treating” Aortic Valve Calcification

**DOI:** 10.3390/jcdd10030096

**Published:** 2023-02-23

**Authors:** Donatos Tsamoulis, Iliana Siountri, Loukianos S. Rallidis

**Affiliations:** 1 1st Department of Internal Medicine, Thriasio General Hospital of Eleusis, 192 00 Athens, Greece; 2 Society of Junior Doctors, 5 Menalou Str., 151 23 Athens, Greece; 3 1st Department of Internal Medicine, General Hospital of Nikaia “Agios Panteleimon”, 184 54 Nikaia, Greece; 4 Second Department of Cardiology, National & Kapodistrian University of Athens, School of Medicine, University General Hospital ATTIKON, 124 62 Athens, Greece

**Keywords:** lipoprotein(a), aortic valve stenosis, aortic valve calcification, familial hypercholesterolemia, statins, PCSK9 inhibitors, lipoprotein apheresis, RNA-related treatments, olpasiran, pelacarsen

## Abstract

The treatment of patients with aortic valve calcification (AVC) and calcific aortic valve stenosis (CAVS) remains challenging as, until today, all non-invasive interventions have proven fruitless in preventing the disease’s onset and progression. Despite the similarities in the pathogenesis of AVC and atherosclerosis, statins failed to show a favorable effect in preventing AVC progression. The recognition of lipoprotein(a) [Lp(a)] as a strong and potentially modifiable risk factor for the development and, perhaps, the progression of AVC and CAVS and the evolution of novel agents leading in a robust Lp(a) reduction, have rekindled hope for a promising future in the treatment of those patients. Lp(a) seems to promote AVC via a ‘three hit’ mechanism including lipid deposition, inflammation and autotaxin transportation. All of these lead to valve interstitial cells transition into osteoblast-like cells and, thus, to parenchymal calcification. Currently available lipid-lowering therapies have shown a neutral or mild effect on Lp(a), which was proven insufficient to contribute to clinical benefits. The short-term safety and the efficacy of the emerging agents in reducing Lp(a) have been proven; nevertheless, their effect on cardiovascular risk is currently under investigation in phase 3 clinical trials. A positive result of these trials will probably be the spark to test the hypothesis of the modification of AVC’s natural history with the novel Lp(a)-lowering agents.

## 1. Introduction

Aortic valve calcification (AVC) is the most common valvular disease, affecting approximately 30% of the population over 65 years of age. The prevalence of calcific aortic valve stenosis (CAVS) exceeds 10% in people over 75 years of age, while severe disease affects more than 3% of the elderly [1]. Given the unfavorable prognosis of the disease and the lack of efficient non-invasive methods to modify the disease’s natural history, AVC and CAVS remain a major public health concern [2].

To date, no pharmaceutical agent has been shown to improve or slow progression of the disease, and the only interventions available for patients with severe and symptomatic CAVS are surgical aortic valve replacement (SAVR) and transcatheter aortic valve implantation (TAVI).

**Figure 1:**
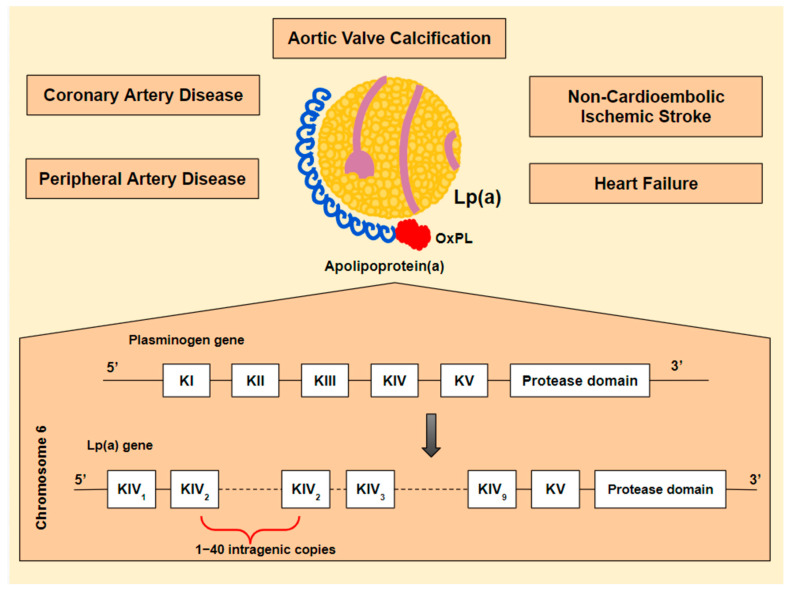
Lipoprotein(a) [Lp(a)] is an atherogenic, pro-inflammatory and, potentially, thrombogenic lipoprotein. These properties are mostly determined by its composition: a newly synthesized low-density lipoprotein (LDL) particle, linked to a unique apolipoprotein, known as apolipoprotein(a) [apo(a)] [7]. What makes apo(a) specific is its structural similarity to plasminogen and its binding to oxidized phospholipids (OxPL). The Lp(a) gene is believed to be derived from the plasminogen gene (PLG). PLG encodes five kringle domains (KI-KV), of which only KIV and KV are maintained in Lp(a). Additionally, KIV is converted, resulting in ten different KIV subtypes from which KIV-2 is found in multiple intragenic copies (1–40). Its similarity to plasminogen could theoretically lead to thrombogenicity [8,9,10]. The LDL particle contributes, partly, to Lp(a) atherogenic potential [11], while OxPL may play a major proinflammatory role [12,13,14].

The recognition of lipoprotein(a) [Lp(a)] as a strong and potentially modifiable risk factor for the development of CAVS brought CAVS treatment to the forefront [3]. Lp(a) is associated with increased cardiovascular risk, including coronary artery disease (CAD), peripheral artery disease (PAD), heart failure, CAVS, and non-cardioembolic ischemic stroke [3,4,5,6] (Figure 1). Until recently, hypolipidemic agents had no or mild effect on Lp(a) levels. However, new emerging agents, targeting Lp(a) synthesis specifically, are showing significant effectiveness and have rekindled hope for a promising future in the treatment of patients with high Lp(a) levels. The effect of these agents on AVC and disease progression is yet to be investigated.

The aim of this review is to examine the association between Lp(a) and CAVS and the effect of currently available lipid-lowering therapies (LLTs) on Lp(a). It will also present the preliminary outcomes of mRNA related therapies targeting Lp(a) and their potential role in reducing cardiovascular events as well as in modifying AVC’s natural history.

## 2. Association of Lp(a) with Aortic Stenosis

Particular research is being conducted on the correlation of CAVS or AVC with Lp(a). AVC, also known as aortic sclerosis, is defined as the non-obstructive calcification of the aortic valve (aortic jet velocity ≤ 2.5 m/s), and is considered to be a precursor of CAVS (aortic jet velocity > 2.5 m/s). The two entities are believed to be different stages of the same pathology. Both AVC and CAVS are characterized by an age-related incidence and an increasing prevalence in the general population because of population aging.

AVC and CAVS were believed to be the result of a passive, age-related process secondary to valvular damage, known as the ‘wear and tear’ mechanical stress theory. However, recent studies suggest that AVC is an active process, similar to atherosclerosis [15].

Lipid deposition, macrophage infiltration, extracellular matrix (ECM) dysregulation including collagen overproduction, and osteoblastic transitioning of valve interstitial cells (VICs) comprise the disease’s pathophysiology [15].

Age, male gender, low-density lipoprotein-cholesterol (LDL-C), arterial hypertension, and smoking are well described risk factors for the development of CAVS [16], while bicuspid aortic valve (BAV) is strongly associated with earlier disease development and faster disease progression [17]. Lp(a) constitutes a novel recognized risk factor for the development of AVC, with the first studies suggesting a potential association published in the late 1990s [16,18].

### 2.1. Lp(a) and AVC in the General Population

Currently, the crucial and causal role of Lp(a) in the AVC is well established, mainly via Mendelian randomization analyses conducted in general population studies. Combined data from two large prospective population studies, the Copenhagen City Heart Study and the Copenhagen General Population Study indicate a linear association of Lp(a) levels with the risk of a future diagnosis of valve calcification or stenosis. In particular, Lp(a) seems to be an independent risk factor for the development of AVC, even in patients with levels between the 67th and the 89th percentile (20 to 64 mg/dL) (1.6 [95% CI: 1.1 to 2.4]), while in those with levels above the 95th percentile (>90 mg/dL), an almost three-fold increased risk is observed [19].

Moreover, in the EPIC-NORFOLK prospective study, patients with Lp(a) > 50 mg/dL had a two-fold higher risk for the development AVC (2.15 (1.39–3.32), *p* = 0.001) [20].

### 2.2. Lp(a), AVC/CAVS in Patients with Heterozygous Familial Hypercholesterolemia

The increased prevalence of AVC and CAVS in patients with familial hypercholesterolemia (FH) has also been investigated since the late 1990s [21,22]. At this time, the defining role of Lp(a) in disease prevalence and progression was yet to be established.

FH is a common autosomal (prevalence of heterozygous FH [heFH] 1 in 250], dominantly inherited disorder of cholesterol metabolism caused by mutations in the LDL receptor (LDLR) gene, the apolipoprotein-B (apoB) gene or the proprotein convertase subtilisin/kexin type 9 (PCSK9) gene. FH is characterized by elevated LDL-C levels which are almost two times higher in heterozygous subjects compared to the general population. In parallel, patients with FH seem to have elevated Lp(a) levels compared to non-FH subjects, potentially due to decreased Lp(a) clearance [23]. AVC seems to be almost two times more frequent in heFH patients compared to the general population, while non-LDLR mutations seem to be even more predictive of AVC compared with LDLR mutations. As both FH and high Lp(a) levels are strongly associated with an increased prevalence of AVC and aortic stenosis (AS), a two-shot hypothesis is suggested in those subjects [24].

Vongpromek et al. reported a significant association between Lp(a) levels and AVC in 129 asymptomatic heFH patients treated with statins. In this study, AVC was detected and quantified by computed tomography (CT) and Lp(a) levels > 50 mg/dL were an independent predictor of AVC. Interestingly, Lp(a) was not associated with coronary artery calcification (CAC), suggesting a different pathophysiology of these two entities [25,26]. This is also supported by a recent study showing that Lp(a) ≥ 70 mg/dL in 191 patients with stable CAD was specifically associated with the progression of low-attenuation and fibro-fatty plaques, but not with plaque calcification [27].

### 2.3. Lp(a), AVC/CAVS in Patients with Bicuspid Aortic Valve

Bicuspid aortic valve (BAV), found in ~1% of the general population, is strongly associated with early AVC and AS. The majority of patients under 65 years old presenting with AS have a BAV, illustrating, therefore, the significance of aortic valve malformations in disease development [17].

The accelerated pattern of AVC in BAV offers a good model to investigate the association of AVC with Lp(a) levels. However, there is very little data on this field. A case-control study conducted by Sticchi et al., including 69 subjects with ultrasound evaluated BAV, demonstrated a statistically significant correlation between Lp(a) levels and AVC prevalence and severity in BAV patients [28].

### 2.4. Lp(a) and AVC/CAVS; Causation

Even if Lp(a) association with AVC is evident, its causative link to the disease remained in question until the determination of a specific single-nucleotide polymorphism (SNP) on the Lp(a) gene, namely the rs10455872 variant, which was associated with elevated Lp(a) levels as well as with the presence of clinically important AVC. In a study by Thanassoulis et al., the presence of the allele rs10455872 was associated with a two-fold increase in AVC detected by Computed Tomography (CT), even after adjustments for other known risk factors [29], suggesting a causal association of Lp(a) with the disease.

### 2.5. Lp(a) and Progression of AVC/CAVS; an Association in Question

Elevated Lp(a) levels may be associated not only with disease incidence but also with faster disease progression and worse prognosis.

The ASTRONOMER trial constitutes a landmark on the research of Lp(a)-mediated AVC. Two hundred and twenty patients with mild to moderate CAVS were followed up using annual Doppler echocardiography and measurement of peak aortic valve velocity for a median period of 3.5 years. Patients in the top tertile of Lp(a) levels (>58.5 mg/dL) demonstrated faster disease progression compared to patients in the bottom two tertiles (<58.5 mg/dL) [peak aortic valve velocity: +0.26 ± 0.03 vs. +0.17 ± 0.02 m/s/year; *p* = 0.005], while they presented with a two-fold higher rate of clinical events, including Aortic Valve Replacement (AVR) and death. These endpoints were not affected by the presence of BAV or other AVC risk factors. In the same study, age is underlined as a determinant factor. Patients <57-year-old belonging to the top tertile had a twofold increase in disease progression compared with patients in the bottom tertile, while in those over 57 years old, Lp(a) contribution on CAVS progression was not statistically significant [14]. Even more, a secondary analysis of the ASTRONOMER trial suggests a linear association between Lp(a) levels and CAVS progression [30].

Another study, conducted by Zheng et al., using positron emission tomography (PET)-CT imaging and echocardiography follow-up and including data from two prospective multimodality imaging studies (SALTIRE and RING OF FIRE studies), came to confirm the ASTRONOMER trials’ conclusion on Lp(a)’s linear association with CAVS progression. What this study adds to our knowledge is that a significantly faster progression of AVC as well as a hemodynamic deterioration and a higher event rate were observed in an older study population, with a mean age of 70 years old. This observation is in contrast with ASTRONOMER’s suggestion of an age cut-of effect of Lp(a) on AVC, while a lower threshold of 35 mg/dL for Lp(a) levels was established, resulting in an almost two-fold increase in peak jet velocity [31].

A recent study by Kaiser et al. came to contest the correlation of Lp(a) levels with AVC progression. In this study, 922 subjects recruited from the population-based Rotterdam Study were followed-up for a median period of 14 years using CT imaging. The results confirmed the association of baseline Lp(a) levels with the subsequent development of AVC in patients without AVC at baseline. However, in those with a preexisting AVC, disease progression was independent of Lp(a) levels. These results suggest that any emerging Lp(a) lowering treatment may be efficient only in the pre-sclerosis phase [32].

### 2.6. Pathophysiology

The mechanisms by which Lp(a) contributes to AVC and AS remain under investigation. AVC is believed to be a procedure similar to atherosclerosis. Inflammation, lipid deposition, fibrosis and calcification compose the histologic features observed in both entities. Calcification seems to be a key factor in both AVC and atherosclerosis. However, the pathophysiology of calcification differs between the two entities and AVC seems to be independent of atheroma calcification [33,34].

Calcification of the aortic valve is the result of a cell differentiation process. Specifically, VICs, the predominant population of cells in the aortic valves, are characterized by excessive plasticity and the potential to differentiate into osteoblast-like cells as a result of various stimuli. In vitro studies suggest a determinant role of Lp(a) on VIC transition toward osteoblast-like cells. Zheng et al. investigated the potential of Lp(a) in inducing osteogenic differentiation of VICs and concluded that one week of exposure of VICs in vitro to high Lp(a) levels leads to a significant increase in pro-inflammatory (interleukin-6 [IL-6]) and pro-osteoblastic transcription factors [bone morphogenetic protein-2 (BMP2), and runt-related transcription factor 2 (RUNX2)]. In addition, the effect of Lp(a) seems to be attenuated after its oxidized phospholipid (OxPL) content inactivation via either monoclonal antibodies targeting OxPL or a specific mutation on apolipoprotein(a) [Apo(a)] abrogating its ability to bind OxPL, thus highlighting a functional role of OxPL [31].

**Figure 2:**
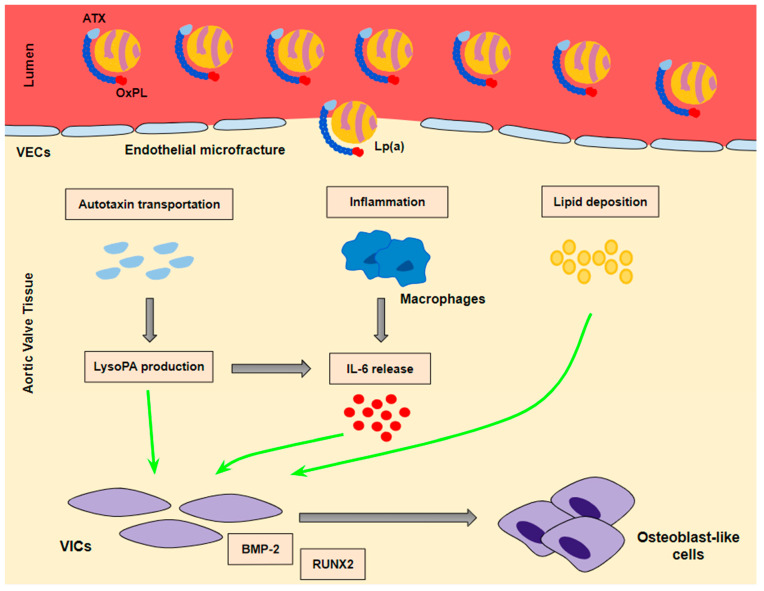
The mechanisms whereby Lp(a) contributes to valve interstitial cell transition into osteoblast-like cells. A ‘three hit’ hypothesis: Lp(a) penetrates into aortic valve tissue through microfractures in the valve endothelium. Autotaxin transportation and inflammation as well as lipid deposition contribute to the transition of VICs into osteoblast-like cells. BMP-2 and RUNX2 are the key signaling factors of the transition process.

Lp(a) seems to lead to VIC differentiation in various ways (Figure 2):

(a) Lp(a) still preserves LDL properties and, thus, significantly contributes to intravalvular lipid deposition. Some studies propose that Lp(a) also has a role in the wound-healing process. Given the mechanical stress to which aortic valve leaflets are continuously submitted and the subsequent microfractures in their structure, Lp(a) may have an essential role in the lipid deposition process conducted via the healing process. Lipid deposition seems to contribute to VIC differentiation via the BMP2 pathway activation [34].
(b) The OxPL content of apo(a) and its pro-inflammatory properties seem to be key factors of AVC. Inflammation is believed to lead to the activation of the calcification process via the activation of an innate immune response. OxPLs exhibit damage-associated molecular patterns (DAMPs) which, through toll-like receptors (TLRs) expressed on the VIC surface and the Nuclear factor kappa B (NF-kB) pathway, lead to the expression of IL-6. In vitro studies have demonstrated that IL-6 has the potential to activate the BMP2 pathway, thus leading to VIC differentiation [34].
(c) Autotaxin, an oxidizing enzyme, may also have a crucial role in VIC transition. By binding to the Lp(a) molecule, autotaxin is transferred to aortic valve leaflets and triggers the oxidative transformation of phospholipid lysophosphatidylcholine (LysoPC) to lysophosphatidic acid (LysoPA). LysoPA promotes the expression of IL-6 and the activation of the BMP2 pathway leading, once again, to VIC differentiation into osteoblast-like cells, and finally to AVC [34,35].

## 3. The Failure of Statins to Affect the Progression of AS

The similarities of AS with the atherogenic process and the association of cholesterol with AS raised the expectation that an intense LLT with a strong statin would be able to reduce the rate of AS progression. This hypothesis was tested in the ASTRONOMER trial, which explored the effect of rosuvastatin 40 mg on CAVS progression in 269 asymptomatic patients with mild to moderate CAVS in recruitment time. All patients had annual echocardiographic assessment of AS progression for a median follow-up period of 3.5 years [36]. Rosuvastatin failed to positively affect the annualized increase in the peak AS gradient and these results were in accordance with those of a previous double-blind, placebo-controlled trial, which also failed to demonstrate any significant effect of atorvastatin on CAVS progression [37].

Statin treatment has also been associated with faster progression of calcification in atheromatous plaques. In the PARADIGM study, a prospective cohort study investigating the effect of statins on plaque characteristics, a faster plaque calcification process was observed in the statin group [38]. Given the similarities between atheromatosis and AVC, a comparable effect of statins on aortic valve leaflets has been proposed. In contrast with coronary artery plaques, where calcification progression seems to have a positive effect as it stabilizes the atheroma, a similar effect on the aortic valve could have a negative impact, increasing leaflet stiffness and promoting stenosis.

## 4. Currently Available Lipid-Lowering Therapies and Their Impact on Lp(a)

There are no currently available LLTs targeting only Lp(a) levels. Therefore, we present the effect of various LLTs, primarily LDL-C lowering therapies, on Lp(a):

1. Lipoprotein apheresis is the most efficient method for reducing Lp(a) levels that is available to date. Studies conducted in patients with FH who underwent LDL apheresis have shown a ~60% reduction of Lp(a) concentration [39,40]. These promising outcomes and the lack of equally sufficient non-invasive approaches explain the fact that in Germany, since 2008, Lp(a) apheresis has been available for patients with elevated Lp(a) levels and established cardiovascular disease (CVD), even without elevated LDL levels [41]. Even if Lp(a) reduction via lipoprotein apheresis seems to be essential, its impact on CVD risk remains under investigation. More useful data is expected from the MultiSELECt trial, a multicenter multinational prospective two-arm matched-pair observational study which is expected to be completed by the end of 2022 [42].

2. Statins have a controversial effect on Lp(a) levels. Despite the initial conviction that statin treatment could decrease Lp(a) levels by augmenting LDLR expression, various studies have demonstrated a neutral or, even, negative effect of statins on Lp(a) levels [43,44,45,46,47,48,49]. A meta-analysis of 3 RCTs showed that statins increased Lp(a) levels by ~10% [48]. Moreover, different types and doses of statins have been proposed to have separate effects on Lp(a) levels. In particular, it has been suggested that atorvastatin has a slightly worse effect on Lp(a) levels compared to other statins, and its effect is dose-dependent, while the effect of pitavastatin seems to be more favorable [49].

3. PCSK9 inhibitors (PCSK9i) also reduce Lp(a) levels to a certain extent. In a systematic review and meta-analysis of phase II and phase III RCTs including evolocumab and alirocumab in comparison to placebo and/or ezetimibe and/or other LLT, a ~27% reduction of Lp(a) levels was observed [50]. At first, it was believed that Lp(a) reduction due to PCSK9i is achieved exclusively via LDLR up-regulation. However, a pooled analysis from 10 ODYSSEY Phase 3 studies indicated a high and unexplained discordance between LDL-C and Lp(a) reduction in patients treated with a PCSK9i. If LDLR up-regulation could fully explain Lp(a) reduction due to PCSK9i, then LDL-C and Lp(a) reduction should follow a similar pattern. The absence of such a pattern suggests the presence of additional mechanisms [51]. It is therefore believed that Lp(a) reduction due to PCSK9i administration is achieved by a dual mechanism including both increased Lp(a) catabolism and decreased Lp(a) production [52]. LDLR up-regulation could explain the increased catabolism of Lp(a) particles [53], while in contrast, the inhibition of Lp(a) expression due to PCSK9i is less understood [54].

4. Niacin and cholesteryl ester transfer protein (CETP) inhibitors have also been shown to decrease Lp(a) by ~20% and 30%, respectively [55,56]. However, both medications failed to show a clinical benefit in patients with CAD in the era of statins [57,58].

**Figure 3:**
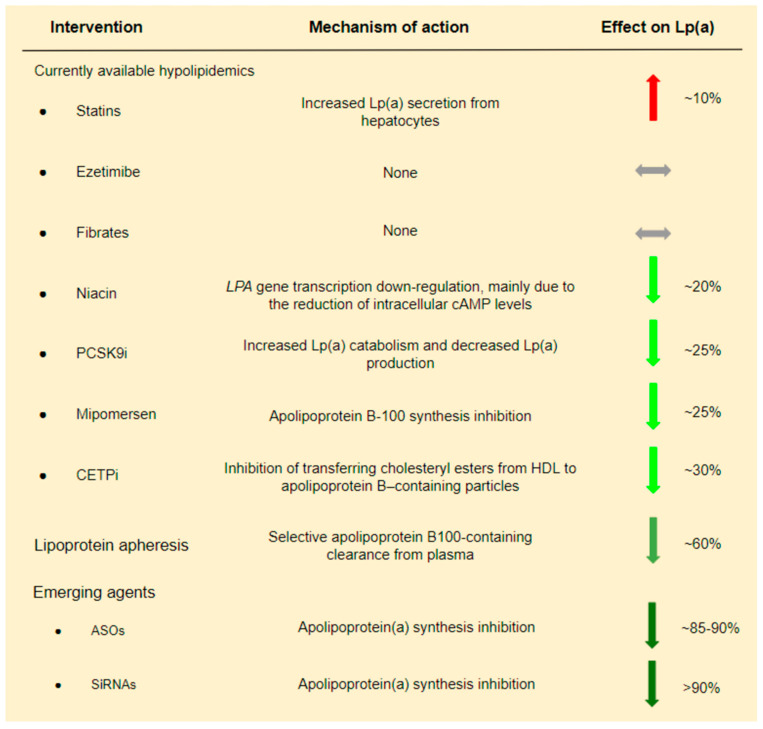
Current and emerging lipid lowering treatments and their effect on Lp(a).

In conclusion, from currently used hypolipidemic drugs, only PCSK9i modestly decreases Lp(a) (Figure 3). Analysis of the FOURIER Trial suggests that Lp(a) reduction by evolocumab possibly contributes to the clinical benefit. In particular, it was shown that individuals with higher baseline Lp(a) levels had greater absolute Lp(a) reductions with evolocumab, and this was associated with greater coronary risk reduction [59]. These findings are encouraging and somehow in contrast to a Mendelian randomization analysis which proposed that a very large Lp(a) decrease, i.e., ~100 mg/dL, is required to have a similar CVD risk reduction with an LDL-C reduction of 39 mg/dL [60].

## 5. Emerging Treatments

A fundamentally novel approach is the use of agents that target RNA in order to interfere with the synthesis of Lp(a) through intracellular pathways, leading to gene silencing. Currently, there are two types of oligonucleotide-mediated gene silencing agents being investigated: “antisense” oligonucleotide-based molecules (ASOs) and small interfering RNAs (siRNAs). Both approaches act by creating an “antisense” oligonucleotide-based strand, which binds to its cognate RNA in vivo and inhibits apo(a) synthesis. Their main difference is that ASOs are single stranded, while siRNAs are double stranded, containing a sense and an antisense strand. The pharmacologically active half is the antisense strand, while the sense strand acts as a ‘‘drug delivery device’’ [61].

The concept of this treatment was first documented with the use of mipomersen, an FDA-approved ASO inhibitor of apoB-100 synthesis. Mipomersen acts by directly inhibiting apoB-100 synthesis and, thus, LDL plasma concentration. Mipomersen has been used in adults and pediatric populations with homozygous and heFH, already treated with maximally tolerated statin treatment or plasmapheresis [62,63]. A pooled analysis of 382 patients with FH or severe polygenic hypercholesterolemia demonstrated a significant 26% reduction of Lp(a) in the mipomersen arm [62]. The mechanism proposed by the authors is that the inhibition of apoB-100 synthesis results in limited apoB-100 particle availability for the formation of Lp(a). This hypothesis is also supported by non-human studies where mipomersen resulted in reduced apoB-100 containing particles while hepatic apo(a) mRNA and apo(a) plasma concentration remained stable [64,65].

The first specific ASO directly targeting Lp(a) synthesis, ASO 144367, was investigated in transgenic mouse models. The results were revolutionary, showing for the first time that a pharmaceutical agent can drastically reduce Lp(a) levels and their associated OxPL levels up to 86% and 93%, respectively [66].

Following the promising results from ASO 144367, multiple ASOs were screened for their ability to reduce apo(a) expression in vitro. ISIS-APO(a)Rx, also known as IONIS-APO(a)Rx, was identified as optimal for further investigation in non-human and human trials [66].

In a phase I clinical trial of IONIS-APO(a)Rx in healthy adults, a single-dose (50 mg, 100 mg, 200 mg, or 300 mg or placebo) as well as a multi-dose (100 mg, 200 mg, or 300 mg or placebo at days 1, 3, 5, 8, 15, and 22) subcutaneous injection schedule was followed. Single-dose administration did not reduce Lp(a) levels significantly. In contrast, multi-dose administration resulted in a dose-dependent Lp(a) reduction (100 mg 39.6%, *p* = 0.005 vs. placebo; 200 mg 59%, *p* = 0.001 vs. placebo; 300 mg 77.8%, *p* = 0.001 vs. placebo) on day 36. Lp(a) reduction was sustained almost 3 months later [67]. This positive effect was also demonstrated in the subsequent phase II clinical trial. The population of the study was divided into two cohorts based on baseline Lp(a) levels (125–437 nmol/L in cohort A; ≥438 nmol/L in cohort B). Following a multi-dose subcutaneous injection schedule (100 mg, 200 mg, and then 300 mg, once a week for 4 weeks each vs placebo) a significant reduction of Lp(a) levels was observed in both cohorts [66.8% (SD 20.6) in cohort A and 71.6% (SD 13.0) in cohort B (both *p* < 0.0001 vs. pooled placebo)] almost 3 months later [68,69].

In order to optimize ASO hepatocyte endocytosis, IONIS APO(a)-LRx, an ASO conjugated with N-acetylgalactosamine (GalNAc), was designed. GalNAc is a molecule that binds to the asialoglycoprotein receptor and that is highly expressed on hepatocytes, resulting in rapid endocytosis and therefore improving drug potency and tolerability. IONIS APO(a)-LRx was, therefore, investigated in a phase 1/2a first-in-man trial using lower doses compared to IONIS APO-Rx trials, and resulting in even better effectiveness (66% in the 10 mg group, 80% in the 20 mg group, and 92% in the 40 mg group (*p* = 0.0007 for all vs. placebo) at day 36. Treatment with both IONIS-APO(a)Rx and IONIS-APO(a)-LRx, significantly reduced OxPL and OxPL-apoB, proinflammatory particles that promote atherogenesis as well as calcification of the aortic valve [68,69].

Lastly, an ongoing phase III randomized, placebo-controlled clinical trial (Lp(a)HORIZON Trial) will assess the effect of IONIS-APO(a)-LRx (pelacarsen) on major cardiovascular events in patients with established CVD and Lp(a) ≥70 mg/dL. Primary endpoints include time to first occurrence of major adverse cardiovascular events. The trial currently has 8221 patients participating, and it is expected to end in 2025 [70].

Following ASOs, a second emerging intervention directly targeting Lp(a) production is the use of siRNAs in order to inhibit apo(a) mRNA expression. Studies in non-human primates showed a robust reduction of plasma Lp(a), over 95%, after a single dose of the agent, which was sustained over a 3-week follow-up period [71]. Subsequent human phase I studies were conducted in patients with elevated Lp(a) levels and without known CVD. SLN360, an siRNA targeting apo(a) mRNA linked with GalNac, was used in ascending doses, and a dose-dependent and persisting (over 150 days) reduction of Lp(a), up to 98% was demonstrated [72]. SLN360 is currently investigated in a multi-center, randomized, double-blind, placebo-controlled, phase II study, expected to be completed by the end of 2024 [73].

Another siRNA called olpasiran (AMG 890), has also been investigated in human trials. In a phase I placebo-controlled clinical trial of adults with elevated Lp(a) plasma levels, the agent was well tolerated, and a 71–97% reduction in Lp(a) concentration was demonstrated [74]. Recently, the phase II clinical trial of olpasiran was completed, showcasing a robust reduction in Lp(a) levels (placebo-adjusted mean percent changes of −70.5% with the 10-mg dose, −97.4% with the 75-mg dose, −101.1% with the 225-mg dose administered every 12 weeks, and −100.5% with the 225-mg dose administered every 24 weeks) which is dose-dependent and sustained almost one year after the first subcutaneous injection [75]. After the successful completion of the phase II clinical trial, olpasiran is currently investigated in a phase III (OCEAN(a)—Outcomes Trial), randomized, double-blind, placebo-controlled study which is estimated to be completed by the end of 2026 [76] (Table 1).

## 6. Novel Lp(a)-Lowering Therapies: The Dawn of Pharmaceutical Treatment of AS?

The well documented association of Lp(a) with the fibro-calcific remodeling of the aortic valve suggests that Lp(a) might be a good target to prevent AVS. Therefore, it is tempting to speculate that the novel Lp(a)-lowering therapies might be able to prevent AVC or to decelerate the progression of AVC to CAVS. However, there is currently no randomized controlled trial testing this hypothesis, and there are several issues that arise under this perspective:

(1) What is the best technique to assess the effectiveness of Lp(a)-lowering agents? Echocardiography is an excellent tool to assess AVS, but it is unable to quantitate AVC. On the contrary, CT can measure AVC and can objectively evaluate the effectiveness of the Lp(a)-lowering treatments which will possibly exert their beneficial effect by delaying the calcification process.
(2) Lp(a)-lowering agents should be ideally tested after the completion of phase III trials which explore their safety and effectiveness on cardiovascular events.
(3) How early should Lp(a)-lowering agents be given? “The earlier, the better” principle of statins administration in acute coronary syndrome is likely to be applicable in the setting of AVC. There is data suggesting that the impact of Lp(a) on the progression of calcification may be weak once AVC has been initiated [27].
(4) What is the target population that is more likely to benefit from the new pharmaceutical interventions? It is appropriate to test the new Lp(a)-lowering therapies in populations at high risk of developing AVC/CAVS, i.e., patients with high Lp(a) levels and BAV, or possibly heFH patients? Particularly, in the case of heFH, by lowering Lp(a) it is possible to obtain a dual beneficial effect, i.e., a reduction of cardiovascular events and the prevention of AVC/CAVS.
(5) What will be the threshold Lp(a) levels which will justify a pharmaceutical intervention? In the Lp(a)HORIZON trial, pelacarsen is tested in patients with established CVD and Lp(a) ≥ 70 mg/dL. For the prevention of AVC/CAVS it might be sensible to set higher Lp(a) cutoff levels.

All of these issues comprise a complex puzzle towards the future perspective for the conservative treatment of AVC/CAVS. The topic is getting more complicated by considering the complex pathophysiology of AVC/CAVS, where Lp(a) is only one significant player. We think that the successful completion of phase III trials is a prerequisite before Lp(a)-lowering treatments will be tested in the context of aortic valve disease. It will be reasonable to initially test this hypothesis in patients with very high Lp(a) levels, aortic valve disease in pre-calcific stages, and probably in those with coexistent BAV or heFH. All of these suggest that it will be several years before this research hypothesis can be answered.

## 7. Conclusions

What makes the Lp(a) and AVC/CAVS correlation special is the emerging hope that in the foreseeable future there may be novel pharmaceutical agents that will modify the natural history of the disease by targeting and decreasing Lp(a) levels. Currently, no pharmaceutical agent has shown a slowing effect on AVC/CAVS. Novel treatments, namely ASOs and siRNA, have an impressive Lp(a)-lowering effect, and their clinical efficacy in reducing cardiovascular events is currently investigated by the Lp(a)HORIZON Trial, expected to be completed in 2025, and the OCEAN(a)-Outcomes Trial, expected to be competed in 2026. A positive result of these trials may be the spark needed to test the hypothesis regarding the non-invasive modification of AVC’s natural history with novel Lp(a)-lowering agents. The main issues that have to be addressed before testing this hypothesis are the specific characteristics of the target population, the Lp(a) threshold levels, and the optimal timing of treatment.

## Figures and Tables

**Table 1:** Novel mRNA related therapies targeting lipoprotein(a).

Agent	NCT	Intervention	Participants	Study Type	Primary Endpoint	Results	Status
**IONIS-APO(a)Rx** Type of gene silencing: ASO	02160899	SC administration in multiple doses and intervals	64 patients Cohort A: Lp(a) ≥ 50 and <175 mg/dL Cohort B: Lp(a) ≥ 175 mg/dL	Randomize, Double Blind, Placebo-Controlled, Dose Titration, Phase II Trial	1. Percent Lp(a) reduction (at days 85 and 99) 2. Number of TEAEs (at 32 weeks)	1. Lp(a) reduction up 71.6% 2. None TEAE reported	Completed in November 2015
**IONIS APO(a)-LRx (AKCEA-APO(a)-LRx, TQJ230, Pelacarsen)** Type of gene silencing: ASO	04023552 (Lp(a) HORIZON-trial)	80 mg SC administration monthly	8324 patients between 18 and 80 years old with Lp(a) > 70 mg/dL and established ASCVD	Randomized Double-blind, Placebo-controlled, Multicenter, Phase III Trial	Time to first occurrence of clinical endpoint committee confirmed expanded MACE	Not yet available *[In phase II study; Lp(a) reduction up to 92% with 40 mg SC in ascending-doses]*	Expected to be completed in May 2025
**SLN360** Type of gene silencing: SiRNA	05537571	A single SC injection in multiple doses	160 patients between 18 and 80 years old at high risk of ASCVD events and Lp(a) ≥125 nmol/L	Multi-centre, Randomised, Double-blind, Placebo-controlled, Phase II Study	Time averaged change in Lp(a) from baseline at 36 weeks	Not yet available *[In phase I study; Lp(a) reduction up to 98% with 600 mg single dose SC injection. Reduction highly maintained on day 150]*	Expected to be completed in November 2024
**Olpasiran (AMG 890)** Type of gene silencing: SiRNA	05581303 (OCEAN(a)—Outcomes Trial)	SC injection once Q12W	6000 patients between 18 and 85 years old with established ASCVD and Lp(a) ≥200 nmol/L during screening	Double-blind, Randomized, Placebo-controlled, Multicenter Phase III Study	Time to CHD death, myocardial infarction, or urgent coronary revascularization, whichever occurs first	Not yet available *[In phase II study; Lp(a) reduction up to 101.1% with the 225-mg dose administered every 12 weeks (placebo-adjusted mean percent changes)]*	Expected to be completed in December 2026

Abbreviations: Lp(a); Lipoprotein(a), TEAEs; Treatment-Emergent Adverse Events, SC; Subcutaneously, Q12W; every 12 weeks, MACE; Major Adverse Cardiovascular Events, CHD; Coronary Heart Disease, ASCVD; Atherosclerotic Cardiovascular Disease, ASO; “antisense” oligonucleotide-based molecule, SiRNA; small interfering RNA.

## Data Availability

Not applicable.

## Abbreviations

ASOs	Antisense oligonucleotides
AS	Aortic Stenosis
ATX	Autotaxin
AVC	Aortic Valve Calcification
BMP2	Bone morphogenetic protein-2
CAVS	Calcific Aortic Valve Stenosis
CETP	Cholesteryl ester transfer protein
CT	Computed Tomography
CVD	Cardiovascular Disease
HDL	High density lipoprotein
IL-6	Interleukin-6
K	Kringle domain
LDL	Low-density lipoprotein
Lp(a)	Lipoprotein(a)
LysoPA	Lysophosphatidic acid
OxPL	Oxidized phospholipids
PCSK9i	Proprotein convertase subtilisin/kexin type 9 inhibitors
RUNX2	Runt-related transcription factor 2
siRNAs	Small interfering RNAs
VECs	Valve endothelial cells
VICs	Valve interstitial cells
